# Yeast Mitochondrial Escape 1-Like as a Mitochondrial Gatekeeper in Aging-Related Cardiovascular Disease

**DOI:** 10.31083/RCM49477

**Published:** 2026-10-21

**Authors:** Zhihao Wan, Xiaorong Li, Xiaoli Zhou, Yongzheng Guo

**Affiliations:** ^1^Department of Cardiovascular Medicine, Cardiovascular Research Center, The First Affiliated Hospital of Chongqing Medical University, 400016 Chongqing, China

**Keywords:** mitochondria, aging, heart failure, mitochondrial dynamics, proteostasis, cardiovascular diseases

## Abstract

Mitochondrial dysfunction is a hallmark of aging and is closely linked to the progression of cardiovascular disease. Yeast mitochondrial escape 1-like (YME1L) is an adenosine triphosphate (ATP)-dependent i-AAA (intermembrane space AAA, AAA=ATPase associated with diverse cellular activities) protease embedded in the mitochondrial inner membrane that maintains mitochondrial homeostasis by regulating proteostasis, cristae architecture, and metabolic adaptation. Notably, YME1L influences mitochondrial fusion–fission dynamics, respiratory chain supercomplex organization, and oxidative phosphorylation efficiency through the regulated processing of optic atrophy protein 1 (OPA1). Accumulating evidence suggests that age-associated impairment of YME1L function contributes to mitochondrial fragmentation, energetic insufficiency, and reduced stress tolerance, particularly in long-lived, high-energy demand tissues. In the cardiovascular system, experimental models indicate that YME1L deficiency disrupts cristae organization, promotes maladaptive metabolic remodeling, and promotes heart failure-like phenotypes. Conversely, preservation of YME1L activity supports mitochondrial integrity and enhances cardiac resilience under hemodynamic stress. In addition to cardiomyocytes, YME1L also supports endothelial mitochondrial homeostasis, thereby linking mitochondrial proteostasis to vascular aging and diastolic dysfunction. Although YME1L has been extensively studied in cancer biology, its relevance to aging-related cardiovascular vulnerability is increasingly recognized, especially in cardio-oncology, where therapy-induced mitochondrial stress may be exacerbated by age-dependent decline in mitochondrial quality control. This review integrates current evidence supporting YME1L as a context-dependent regulator of mitochondrial stress adaptation, with particular emphasis on the roles of YME1L in cardiovascular aging, heart failure with preserved ejection fraction (HFpEF), and stress tolerance. Rather than positioning YME1L as a universal therapeutic target, we discuss how modulating YME1L-associated pathways may help preserve mitochondrial resilience and mitigate age-related cardiovascular dysfunction.

## 1. Introduction

Population aging has profoundly reshaped the global cardiovascular disease burden. Aging is now recognized not only as a chronological process but also as a major biological determinant of cardiovascular vulnerability, contributing to the development of heart failure, vascular dysfunction, metabolic remodeling, and impaired stress adaptation [1,2]. Among age-associated cardiovascular syndromes, heart failure with preserved ejection fraction (HFpEF) has emerged as one of the most rapidly increasing clinical challenges, characterized by complex interactions among metabolic dysfunction, chronic inflammation, endothelial impairment, and reduced organ resilience [3,4]. Despite substantial advances in cardiovascular medicine, effective therapies capable of reversing or preventing aging-associated cardiac dysfunction remain limited.

Mitochondrial dysfunction is increasingly regarded as a central hallmark of cardiovascular aging. Beyond their canonical role in adenosine triphosphate (ATP) production, mitochondria function as dynamic signaling organelles that regulate calcium homeostasis, redox balance, innate immunity, apoptosis, and metabolic flexibility [5]. In cardiomyocytes and vascular cells, maintenance of mitochondrial quality is particularly critical because of the high energetic demand and limited regenerative capacity of these tissues. Aging-associated mitochondrial abnormalities—including impaired oxidative phosphorylation, excessive reactive oxygen species (ROS) production, mitochondrial DNA (mtDNA) instability, and disrupted mitochondrial dynamics—contribute directly to progressive myocardial and vascular dysfunction [6].

Mitochondrial proteostasis has recently attracted growing attention as an essential mechanism preserving mitochondrial integrity under physiological and pathological stress conditions. The mitochondrial quality control network consists of coordinated systems regulating protein import, folding, degradation, mitophagy, and structural remodeling [7]. Among these regulatory components, ATP-dependent ATPases associated with diverse cellular activities (AAA+) proteases play pivotal roles in eliminating damaged proteins and maintaining mitochondrial membrane architecture [8]. YME1L, an ATP-dependent i-AAA protease localized to the inner mitochondrial membrane, has emerged as an important regulator of mitochondrial proteostasis and stress adaptation.

Importantly, the biological consequences of YME1L dysregulation appear to be highly context dependent. In the cardiovascular system, impaired YME1L activity has been linked to myocardial remodeling, endothelial dysfunction, and reduced tolerance to hemodynamic and metabolic stress. In contrast, increased YME1L expression has been observed in several malignancies, where it may support tumor metabolic adaptation and survival [9,10]. These findings suggest that YME1L functions not as a simple protective or pathogenic factor, but rather as a regulatory node coordinating mitochondrial adaptability in response to tissue-specific stress conditions (Fig. 1).

**Fig. 1.**
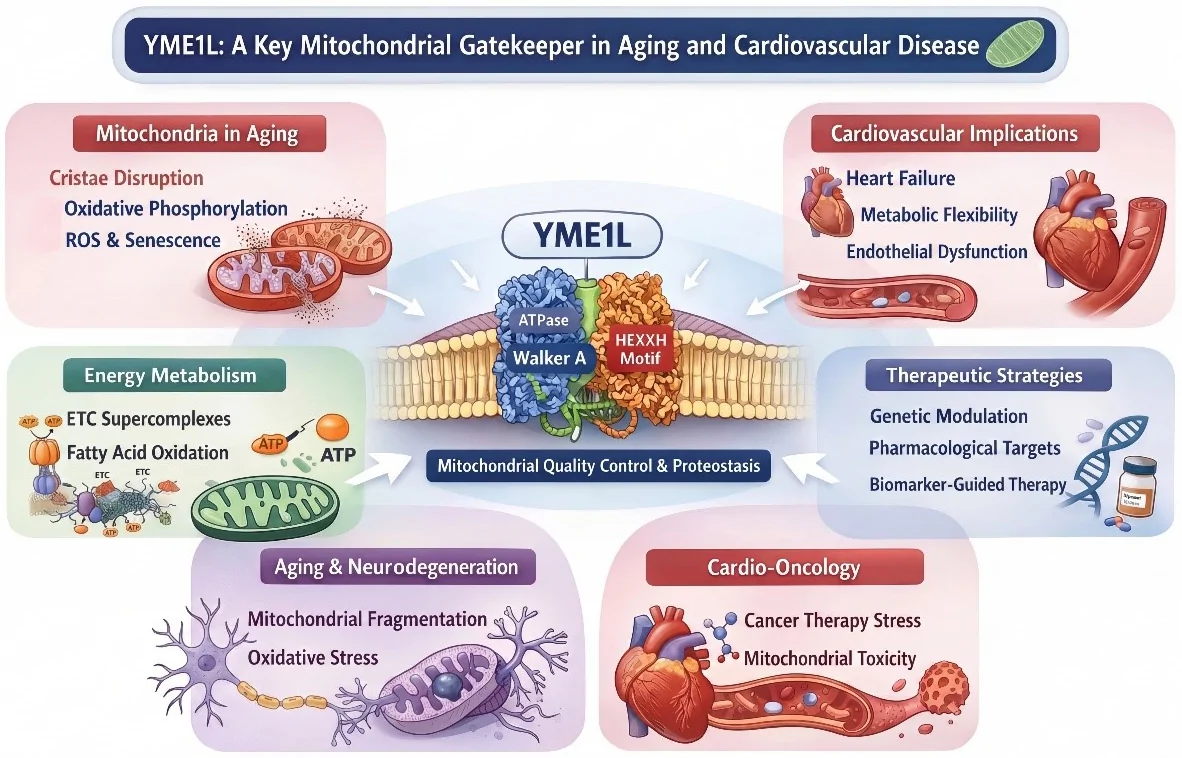
**Yeast mitochondrial escape 1-like (YME1L) as a context-dependent regulator of mitochondrial stress tolerance in aging-related cardiovascular disease**. Schematic overview illustrating the role of YME1L in mitochondrial quality control and its relevance to aging-related cardiovascular dysfunction. YME1L is an ATP-dependent i-AAA protease localized to the mitochondrial inner membrane, where its Walker A ATPase domain and His-Glu-X-X-His zinc-binding motif (HEXXH) metalloprotease motif coordinate proteostasis and cristae organization. Through regulated processing of optic atrophy protein 1 (OPA1), YME1L influences mitochondrial fusion–fission balance, respiratory chain supercomplex stability, and oxidative phosphorylation efficiency. ROS, reactive oxygen species; ETC, electron transport chain; L-OPA1, long isoforms of optic atrophy protein 1; S-OPA1, short isoforms of optic atrophy protein 1; ATP, adenosine triphosphate; i-AAA, intermembrane-space-facing AAA protease, AAA=ATPase associated with diverse cellular activities; HEXXH, His-Glu-X-X-His zinc-binding motif, where X represents any amino acid.

Although YME1L has been extensively investigated in mitochondrial biology and cancer research, its role in cardiovascular aging remains incompletely understood. In particular, the mechanisms linking YME1L-associated mitochondrial proteostasis to HFpEF, vascular inflammaging, and age-related stress intolerance have not yet been systematically summarized. Moreover, the therapeutic feasibility of targeting YME1L-associated pathways in aging cardiovascular disease remains uncertain due to the complexity and tissue specificity of mitochondrial stress responses.

In this review, we summarize current knowledge regarding the structural and biological functions of YME1L, with particular emphasis on its role in mitochondrial dynamics, cristae organization, metabolic adaptation, and cardiovascular aging. We further discuss emerging evidence connecting YME1L dysfunction to heart failure, endothelial injury, inflammaging, and mitochondrial stress signaling, as well as the potential opportunities and limitations of therapeutically targeting YME1L-associated pathways in aging-related cardiovascular disease.

## 2. YME1L Protein: Structural Features and Fundamental Roles in Mitochondrial Homeostasis

### 2.1 Molecular Structure and ATP-Dependent Protease Activity

The mitochondrial i-AAA protease is an ATP-dependent proteolytic enzyme complex whose catalytic domain is located on the inner mitochondrial membrane (IMM) and faces the intermembrane space (IMS) [11,12]. In mammals, the main catalytic subunit of the i-AAA protease is YME1L [11], which is a member of the AAA+ superfamily [11,13]. YME1L assembles into a homohexameric complex in which each subunit contains an AAA+ ATPase domain with a Walker A nucleotide-binding motif and an M41 zinc metalloprotease domain with a His-Glu-X-X-His (HEXXH) zinc-binding motif [14]. This configuration enables YME1L to participate in mitochondrial protein quality control through ATP-dependent proteolysis of misfolded or damaged substrates [15], including components involved in respiratory chain assembly [14]. In addition to its degradative function, YME1L regulates mitochondrial morphology through proteolytic processing of OPA1, thereby coupling proteostasis to mitochondrial structural organization and bioenergetic function [16].

YME1L is also involved in the process of mitochondrial protein importation. For instance, reactive oxygen species modulator 1 (ROMO1) has been identified as a novel component of the human translocase of the inner mitochondrial membrane 23 (TIM23) complex, which is a crucial component of the mitochondrial presequence translocation mechanism. The absence of ROMO1 leads to abnormal mitochondrial inner membrane (MIM) structure and alterations in the proteolytic processing of YME1L [17]. Furthermore, there exists functional crosstalk between the translocase of the inner mitochondrial membrane 22 (TIM22) complex and the YME1L mechanism, collectively maintaining mitochondrial protein homeostasis and integrity [18].

### 2.2 YME1L and OMA1 Regulate OPA1-Mediated Mitochondrial Fusion and Fission

Optic atrophy protein 1 (OPA1) is a key GTPase located in the inner mitochondrial membrane and is essential for maintaining mitochondrial dynamics, cristae architecture, energy metabolism, and regulation of apoptosis [19]. Its gene is mapped to human chromosome *3q28–q29* [19] and generates at least eight distinct isoforms through alternative splicing [20]. These isoforms are mainly classified into long isoforms (L-OPA1) and short isoforms (S-OPA1). L-OPA1 and S-OPA1 function cooperatively by assembling into hetero-oligomeric complexes, collectively driving efficient fusion of the inner mitochondrial membrane [21]. Their synergy maintains the normal morphology and function of mitochondrial cristae, precisely regulating crista membrane curvature, stiffness, and the configuration of crista junctions (CJs). L-OPA1 is primarily anchored to the inner membrane, whereas S-OPA1 is generated through proteolytic cleavage by proteases such as mitochondrial metalloendopeptidase OMA1 (OMA1) or YME1L. Under basal conditions, YME1L preferentially cleaves the S2 site of OPA1, whereas OMA1 is activated (This activation has been proposed to be triggered by autocatalytic cleavage of the C-terminal region of the protease [22]) under mitochondrial stress (e.g., loss of membrane potential, high temperature, or oxidative stress) and cleaves the S1 site [11,23,24]. It has also been proposed that YME1L and OMA1 undergo mutual degradation in response to injury that depolarizes mitochondria, a process governed by cellular energy status. This may be related to reduced nucleotide levels and/or conformational changes induced by membrane depolarization [22]. In the absence of YME1L, compensatory activation of OMA1 occurs, which partially maintains OPA1 processing but often leads to excessive cleavage, thereby inducing mitochondrial fragmentation and cristae destabilization (Fig. 2). This dual-protease system dynamically adjusts the ratio of long and short OPA1 isoforms, allowing mitochondria to adapt their fusion–fission balance to physiological and pathological cues [16,23,24,25,26,27] In YME1L-deficient settings, compensatory activation of OMA1 partially maintains OPA1 processing but often favors excessive cleavage, predisposing mitochondria to fragmentation and cristae destabilization [11].

**Fig. 2.**
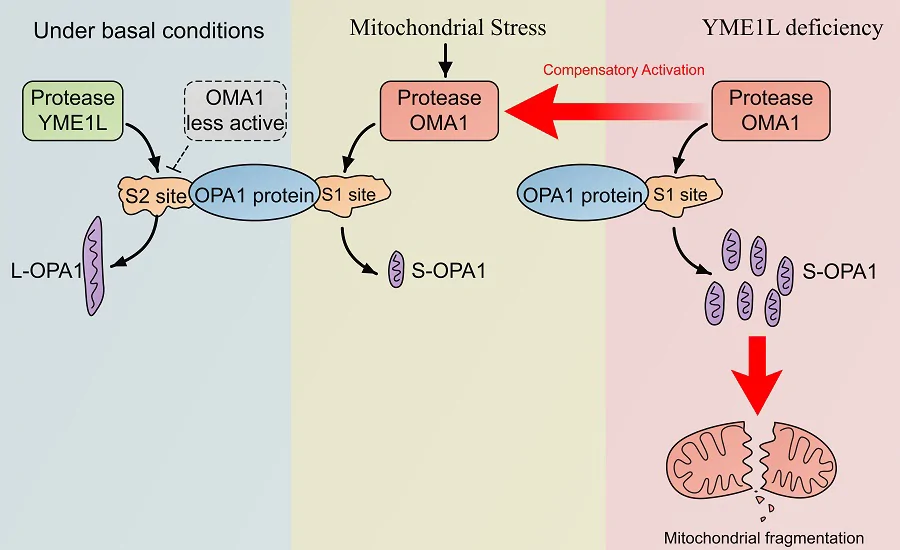
**Processing of OPA1 is coordinately regulated by YME1L and the stress-activated protease OMA1**. Under basal conditions, YME1L preferentially cleaves OPA1 at the S2 site, whereas OMA1 is activated upon mitochondrial stress and responsible for cleavage at the S1 site. This dual protease system dynamically modulates the ratio of long- and short-OPA1 isoforms, enabling mitochondria to adjust their fusion–fission balance in response to physiological and pathological signals. In the setting of YME1L loss-of-function, compensatory activation of OMA1 can partially sustain OPA1 processing but frequently leads to excessive cleavage, thereby driving mitochondrial fragmentation and destabilization of cristae structure. YME1L, Yeast mitochondrial escape 1-like; OMA1, mitochondrial metalloendopeptidase OMA1; OPA1, optic atrophy protein 1.

### 2.3 YME1L Regulates Energy Metabolism

Mitochondrial cristae are structures formed by the invagination of the inner mitochondrial membrane, which significantly increases the surface area of the inner membrane and provides an essential assembly platform and reaction space for oxidative phosphorylation complexes [28,29,30,31,32]. Dynamic changes in cristae, including alterations in their number, length, width, lateral arrangement, curvature/stiffness, and cristae junction geometry, can affect the function of the oxidative phosphorylation system [33].

The F1Fo-ATP synthase oligomers are one of the key protein complexes responsible for shaping mitochondrial cristae [28]. These oligomers induce membrane curvature, driving the formation of the characteristic sheet-like or tubular morphology of cristae [34]. Cardiolipin (CL), a tetra-acyl chain phospholipid, interacts with ATP synthase dimers and is crucial for both the structure and function of mitochondrial cristae. In mitochondrial disorders such as Barth syndrome (BTHS), the cristae membrane accumulates more monolysocardiolipin (MLCL) with reduced unsaturation, leading to impaired cristae structure and function. Molecular dynamics simulation studies have shown that the lipid composition in BTHS affects the conformation of ATP synthase dimers, thereby altering cristae structure [31].

The mitochondrial contact site and cristae organizing system (MICOS) is another crucial protein complex that works in concert with F1Fo-ATP synthase oligomers to control the biogenesis of the mitochondrial inner membrane and the formation of cristae [28]. The MICOS system is responsible for connecting the inner and outer membranes and maintaining the structure of cristae at the crista junctions [35]. These crista junctions act as a barrier, separating the cristae membrane from the inner boundary membrane (IBM), regulating ion movement, and ensuring a potential difference between the cristae and the inner boundary membrane [36].

YME1L is associated with the assembly of the MICOS complex, for instance, Stomatin-like protein 2 (SLP2) serves as a proteolytic center scaffold within the mitochondria, anchoring proteases such as presenilins-associated rhomboid-like protein (PARL). And YME1L [37] SLP2 interacts with mitochondrial contact site and cristae organizing system subunit 13 (MIC13) (a subunit of the MICOS complex). The absence of SLP2 leads to YME1L-mediated proteolysis of MIC26 and significant alterations in mitochondrial cristae structure. Furthermore, the translocation of GCN5-like protein 1 (GCN5L1) into mitochondria is associated with the interaction between YME1L and MIC13, which together regulate mitochondrial cristae formation in white adipocytes and the development of obesity [38]. The curvature and stiffness of the cristae membrane, as well as the geometry of the crista junctions, are precisely regulated by OPA1 [39,40]. By regulating OPA1 processing and cristae architecture, YME1L indirectly influences the assembly and efficiency of respiratory chain super complexes. Experimental models consistently show that YME1L deficiency leads to disorganized cristae and reduced activity of oxidative phosphorylation complexes, particularly complex IV, resulting in impaired mitochondrial respiratory capacity [41].

YME1L expression exhibits marked tissue specificity, which likely underlies its diverse functional consequences across organs. In the heart, relatively high YME1L expression supports cristae integrity and respiratory chain organization, whereas reduced YME1L activity in cardiomyocytes is associated with mitochondrial fragmentation and progressive cardiac dysfunction [14,42]. In contrast, neuronal tissues display lower basal YME1L expression and distinct splicing patterns, which may facilitate mitochondrial remodeling in response to fluctuating synaptic energy demands [42].

These observations suggest that YME1L function is shaped not only by its intrinsic protease activity but also by tissue-specific regulatory networks governing mitochondrial dynamics and metabolism [14].

### 2.4 Regulation of Fatty Acid Metabolism and Genomic Stability

YME1L also contributes to metabolic flexibility by regulating the stability of enzymes involved in fatty acid oxidation, including long-chain acyl-CoA dehydrogenase. Loss of YME1L impairs fatty acid utilization and shifts cellular metabolism toward less efficient energy sources. In neuronal models, YME1L deficiency is associated with increased oxidative stress, accumulation of mitochondrial DNA lesions, and activation of the mitochondrial unfolded protein response. These changes may compromise long-term cellular viability and provide a mechanistic link between impaired mitochondrial proteostasis and age-associated neurodegeneration. Specifically, lipid signaling can drive YME1L-mediated proteolysis by modulating the phospholipid composition of mitochondrial membranes. Inhibition of mechanistic target of rapamycin complex 1 (mTORC1) reduces mitochondrial phosphatidylethanolamine (PE) levels via the lipin 1 (LIPIN1)–cytidine 5′-triphosphate: phosphocholine cytidylyltransferase α (CCTα) axis (LIPIN1–CCTα), thereby activating YME1L protease activity and leading to targeted degradation of protein translocases and metabolic enzymes [43]. This mechanism enables cells to rapidly remodel mitochondrial function in response to hypoxic or nutrient-deficient environments.

### 2.5 Tissue Specificity of YME1L

The effects of YME1L deficiency on energy-sensing pathways such as adenosine monophosphate-activated protein kinase (AMPK) and mechanistic target of rapamycin (mTOR) exhibit tissue heterogeneity. In cardiomyocytes, a decline in ATP levels strongly activates AMPK signaling; whereas in hepatocytes, inhibition of mTORC1 activity can occur through energy-independent mechanisms, potentially involving redox and epigenetic regulation. On one hand, YME1L directly regulates metabolic functions by maintaining mitochondrial structural integrity; on the other hand, it exerts indirect regulatory effects by remodeling stress-responsive signaling pathways. In the heart, insufficient YME1L function can impair myocardial contractility due to compromised energy production; in proliferative tissues such as tumors, aberrant YME1L expression may promote adaptive metabolic reprogramming. In cardiac-specific *Yme1l* knockout mice, altered processing of OPA1 was observed. Loss of YME1L enhanced OMA1-dependent OPA1 processing, leading to increased mitochondrial fission and fragmentation of the mitochondrial network, which resulted in dilated cardiomyopathy and heart failure in mice—these pathological changes were alleviated by *Oma1* ablation [44]. In the nervous system, loss of YME1L similarly impairs mitochondrial morphology and further triggers axonal degeneration [26]. Interestingly, although *Oma1* deletion restored abnormal mitochondrial morphology, it failed to rescue axonal degeneration, indicating that the effects of i-AAA protease loss vary across different tissues [26]. YME1L expression exhibits marked tissue specificity, which serves as an important basis for its functional divergence across different organs. In cardiac tissue, YME1L expression levels are relatively high, supporting the integrity of mitochondrial cristae and the orderly assembly of the respiratory chain; decreased YME1L activity in cardiomyocytes can induce mitochondrial fragmentation and progressive cardiac dysfunction. In contrast, neural tissues exhibit lower basal YME1L expression and possess unique splicing patterns, which are more conducive to mitochondrial remodeling during synaptic energy fluctuations. These findings suggest that YME1L function is determined not only by its intrinsic protease activity but also co-shaped by tissue-specific mitochondrial dynamics and metabolic regulatory networks.

## 3. YME1L in Cardiovascular Disease: From Mitochondrial Decline to Cardiovascular Vulnerability

This section reframes YME1L-centered mitochondrial dysfunction within specific aging-related cardiovascular disease contexts, highlighting its role as an upstream determinant of metabolic inflexibility, stress intolerance, and progressive cardiac dysfunction. YME1L exhibits protective functions in specific tissues such as cardiomyocytes through the coordination of mitochondrial biosynthesis, dynamic remodeling, and metabolic reprogramming [45].

### 3.1 YME1L-Mediated Myocardial Injury and Age-Related Heart Failure

Aging is a major risk factor for the development of cardiomyopathy and the transition from compensatory cardiac remodeling to overt heart failure. Unlike acute injury-induced cardiomyopathy, age-related cardiomyopathy progresses gradually and is closely associated with the accumulation of mitochondrial damage and impairment of quality control mechanisms.

Studies have confirmed that OPA1 mediates mitochondrial fusion, prevents cell death, and is associated with cristae morphogenesis [46]. Its role in the adult heart has been investigated in mice partially deficient for this protein; complete ablation of OPA1 leads to intrauterine death. Compared with wild-type littermates, *Opa1*^+/−^ mice exhibit a 50% reduction in myocardial OPA1 content. Moreover, aside from displaying small, fragmented mitochondria, aged *Opa1*^+/−^ mice show impaired cardiac mitochondrial function [47]. OPA1 mutations also lead to late-onset cardiac dysfunction, with no changes early in life, whereas 12-month-old *Opa1*^+/−^ mice exhibit reduced fractional shortening, cardiac output, and myocardial contractility [48]. In contrast, OPA1-overexpressing mice (*Opa1 tg*) develop physiological cardiac hypertrophy at 9 months of age [49] and even outperform wild-type littermates on a treadmill. Mild OPA1 overexpression also protects the heart from ischemic injury following induced infarction, which is accompanied by degradation of the L-OPA1 in 5-month-old wild-type mice but not in age-matched *Opa1 tg* littermates. Therefore, these studies suggest that inhibitors of OPA1 cleavage may indeed have cardioprotective effects.

Wai et al. [44] demonstrated that YME1L is essential for maintaining normal adult cardiac function. Inhibition of YME1L in adult cardiomyocytes activates OMA1, increases S-OPA1, and consequently promotes mitochondrial fission. As a result, 40-week-old *Yme1l* knockout (KO) mice develop dilated cardiomyopathy (DCM) and heart failure, accompanied by elevated serum levels of cardiac troponin T (a marker of cardiac injury) and evidence of necrotic cell death. *In vivo*, YME1L-deficient hearts also exhibit increased glucose uptake and reduced β-oxidation, a typical metabolic shift seen in failing hearts [44]. Concurrent inhibition of OMA1 and YME1L in double-knockout (*Yme1l*^-/-^*Oma1*^-/-^) mice restores mitochondrial morphology and normal cardiac function, demonstrating that long-form OPA1 is both necessary and sufficient for maintaining cardiac function. Furthermore, a high-fat diet drives cardiac mitochondria from glucose utilization toward fatty acid oxidation, thereby suppressing heart failure and restoring normal lifespan in *Yme1l*^-/-^ mice. The pathway identified by Wai et al. [44] may be relevant to age-related heart failure and could serve as a potential therapeutic target. Modulating (i.e., reducing) OMA1 and YME1L levels through pharmacological or dietary interventions may help prevent or manage heart failure. OPA1 plays an important role in the development of (DCM) and is closely associated with apoptosis, imbalance in mitochondrial dynamics, and alterations in cristae structure [50]. Genetic ablation of YME1L in cardiomyocytes induces a dilated cardiomyopathy–like phenotype characterized by mitochondrial fragmentation, cristae disorganization [51], and progressive decline in oxidative phosphorylation capacity. Brain natriuretic peptide (BNP) can promote OPA1-mediated mitochondrial fusion by activating the cyclic guanosine monophosphate-dependent protein kinase (PKG-STAT3) pathway, thereby protecting the heart from diabetic cardiomyopathy [52]. These abnormalities occur before detectable systolic impairment, implying that disruption of mitochondrial cristae organization and substrate utilization may represent early pathogenic events during cardiac remodeling.

Importantly, YME1L deficiency compromises cardiac stress tolerance. In models of pressure overload and ischemia–reperfusion injury, reduced YME1L expression exacerbates mitochondrial depolarization, calcium dysregulation [23], and reactive oxygen species accumulation, thereby accelerating cardiomyocyte apoptosis and contractile failure. YME1L contributes to cardiac adaptation to pressure overload. In mice subjected to transverse aortic constriction, reduced YME1L expression was associated with enhanced OPA1 cleavage and mitochondrial fragmentation [53]. Cardiac-specific YME1L overexpression increased the L-OPA1/S-OPA1 ratio, increased the mitochondrial respiratory control ratio and cardiac ATP levels [26]. The relevance of these findings to cardiac aging requires further investigation.

### 3.2 YME1L Mediates Vascular Aging and Endothelial Dysfunction

Endothelial mitochondria are increasingly recognized as critical regulators of vascular homeostasis, and their dysfunction exerts a profound impact on vascular aging. Vascular aging, characterized by endothelial dysfunction, elevated arterial stiffness, and impaired microvascular adaptability, participates in the pathogenesis of age-related cardiovascular diseases and HFpEF [54]. Endothelial cells (ECs) are highly dependent on precisely regulated mitochondrial dynamics to maintain the balance among energy production, redox signaling, and nitric oxide (NO) bioavailability. In this context, the OMA1/YME1L axis plays a central role. YME1L regulates mitochondrial dynamics through the processing of OPA1: under normal conditions, it generates the pro-fusion L-OPA1; under stress, impaired YME1L activity together with excessive cleavage by OMA1 leads to the accumulation of short OPA1 (S-OPA1) and mitochondrial fragmentation [55]. In endothelial cells, OPA1-mediated mitochondrial fusion has a well-established protective role. *OPA1* silencing reduces endothelial cell migration and flow-mediated elongation. In isolated perfused resistance arteries, flow-mediated dilation (FMD) is impaired in *Opa1*^+/−^ mice (a model of OPA1 haploinsufficiency) and in EC-specific *Opa1* knockout (*EC-Opa1*) mice. Mechanistically, decreased OPA1 expression promotes excessive production of mitochondria-derived superoxide, thereby compromising endothelium-dependent vasodilation [56]. This oxidative stress, arising from disrupted mitochondrial dynamics, is a critical link connecting dysfunction of the YME1L/OPA1 axis to endothelial injury.

YME1L itself is a stress-sensitive protease. Under oxidative stress—such as the redox imbalance commonly observed in vascular aging or HFpEF—YME1L is rapidly degraded, creating a vicious cycle that impairs mitochondrial function [57]. Its degradation requires the simultaneous fulfillment of two conditions: a reduction in intracellular ATP levels (caused by oxidative stress) and the activation of the inner mitochondrial membrane protease OMA1 (triggered by membrane potential depolarization) [57]. ATP depletion alone or OMA1 activation alone is insufficient to induce substantial degradation; only when both occur concurrently does YME1L undergo massive loss. Following YME1L degradation, its substrates (such as mitochondrial import inner membrane translocase subunit Tim17-A (Tim17A), a critical subunit of the mitochondrial protein import complex) accumulate, impairing mitochondrial protein import and inner membrane proteostasis during cellular stress [58]. Ultimately, loss of YME1L activity reduces the resilience of endothelial cells to subsequent oxidative insults and increases the risk of cell death. This mechanism is particularly critical in the context of vascular aging and HFpEF, because endothelial cells are chronically exposed to low-grade inflammation and metabolic stress, and YME1L degradation may accelerate endothelial dysfunction and microvascular rarefaction.

YME1L dysfunction not only affects proteostasis but also triggers broader cellular stress responses. Studies have shown that YME1L deficiency leads to the accumulation of several substrate proteins related to mitochondrial DNA metabolism (such as solute carrier family 25 member 33 (SLC25A33), cytidine/uridine monophosphate kinase 2 (CMPK2), and nucleoside diphosphate kinase 4 (NME4)), thereby promoting mtDNA release into the cytoplasm [42,59]. The released mtDNA acts as a damage-associated molecular pattern (DAMP) that potently activates the cyclic guanosine monophosphate–adenosine monophosphate synthase (cGAS) and stimulator of interferon genes (STING). The pathway may then be referred to as the cGAS–STING innate immune pathway, inducing the production of type I interferons and various inflammatory cytokines [59]. Notably, the cGAS-STING pathway is one of the key signaling axes driving the senescence-associated secretory phenotype (SASP) [60]. Chronic inflammation represents a common pathological basis for cardiovascular diseases such as atherosclerosis and hypertension, and its persistent activation is partly attributable to pro-inflammatory factors secreted by senescent cells through the SASP [61,62]. Therefore, YME1L deficiency-induced mtDNA release and the subsequent cGAS-STING-dependent inflammatory response [63] may directly promote a SASP phenotype in endothelial cells and vascular wall cells, thereby accelerating vascular aging and the progression of related diseases. These findings suggest that YME1L deficiency may mechanistically couple mitochondrial proteostasis failure to vascular inflammaging and endothelial senescence.

## 4. Therapeutic Targeting of YME1L Signaling Pathways

As a central regulator of mitochondrial dynamics and proteostasis, YME1L has emerged as a potential therapeutic target for aging-related and metabolic diseases. Currently, strategies for modulating YME1L function primarily include: small molecule-targeted regulation and biomarker-guided patient stratification.

### 4.1 Small-Molecule Targeted Regulation of the YME1L-Associated Network

The development of specific drugs directly targeting YME1L is still in the early exploratory stage. The most notable candidate compound is lycorine, a natural alkaloid. In studies on esophageal squamous cell carcinoma (ESCC), researchers identified lysosomal acid phosphatase 2 (ACP2) and YME1L as novel prognostic biomarkers for ESCC. A total of 79 candidate drugs were screened out using the Drug Signatures Database. Molecular docking and molecular dynamics simulations demonstrated that lycorine exhibits stable binding affinity with the prognosis-related genes ACP2 and YME1L. Preliminary evidence indicates that lycorine may possess the potential of targeted inhibition against YME1L [64].

As a core regulatory factor of the mitochondrial quality control network, the pathophysiological basis of the YME1L protease in cardiovascular diseases involves multiple interconnected molecular mechanisms. Accordingly, indirect small-molecule regulation targeting the upstream and downstream regulatory networks of YME1L is also applicable to the treatment of aging-related cardiovascular diseases. YME1L function is tightly coupled with redox homeostasis, cristae remodeling complexes, and the stress-responsive protease network. Targeting its upstream or parallel regulatory factors enables fine-tuning of mitochondrial proteostasis without disrupting core mitochondrial functions. For instance, components of the MICOS complex and mitochondrial acetylation regulators such as GCN5L1 can modulate cristae structure and respiratory efficiency in a YME1L-dependent manner.

Oxidized nicotinamide adenine dinucleotide (NAD+) is a redox cofactor in mitochondrial metabolism, whereas reduced nicotinamide adenine dinucleotide (NADH) donates electrons to respiratory complex I. In mice, nicotinamide mononucleotide (NMN) administration increased myocardial NAD+ levels, prevented their decline during ischemia, and reduced infarct size after myocardial ischemia/reperfusion (I/R) [65]. Furthermore, NAD+ regulates mitochondrial dynamics by activating the deacetylases SIRT1 as sirtuin 1 (SIRT1) and sirtuin 3 (SIRT3). SIRT3 deacetylates and activates the mitochondrial fusion protein OPA1, thereby preserving the integrity of the mitochondrial network [66]. Notably, the mitochondrial inner membrane protease YME1L plays a pivotal role in maintaining mitochondrial morphology and function via modulating OPA1 processing. YME1L deficiency leads to mitochondrial fragmentation and is directly associated with dilated cardiomyopathy and heart failure [67]. NAD+ precursors may synergistically regulate mitochondrial fusion with YME1L-mediated OPA1 processing by enhancing SIRT3 activity.

Autophagy is an essential reparative process for maintaining cellular homeostasis [68]. NAD+ supplementation enhances autophagic flux through SIRT1 activation, thereby alleviating microvascular injury in vascular diseases [67]. In diabetic nephropathy models, YME1L has been confirmed to protect renal tubular cells from senescence by mediating mitophagy [69]. This suggests that NAD+ precursors may cooperate with YME1L-associated quality control mechanisms to eliminate damaged mitochondria more efficiently via activating pathways such as SIRT1/PINK1/Parkin.

mTORC1 precisely modulates the dynamic balance of mitochondrial fission and fusion by regulating the translation of mitochondrial fission process 1 (MTFP1) Under physiological conditions, mTORC1 stimulates MTFP1 translation. MTFP1 expression is associated with fission-promoting phosphorylation and the recruitment of the fission GTPase dynamin-related protein 1 (DRP1) to mitochondria. Treatment with potent mTOR active-site inhibitors such as rapamycin leads to excessive mitochondrial fusion due to reduced MTFP1 translation, a process mediated by eukaryotic translation initiation factor 4E (eIF4E)-binding protein (4E-BP) [70]. Imbalanced mitochondrial dynamics is a crucial pathological hallmark of cardiovascular diseases. Moderate mitochondrial fusion contributes to sustaining energy supply and survival of cardiomyocytes, whereas excessive fission is linked to apoptosis and cellular dysfunction. Studies have shown that rapamycin-induced excessive mitochondrial fusion exerts cytoprotective effects. Decoupling MTFP1 levels from the mTORC1/4E-BP axis abolishes excessive mitochondrial fusion upon mTOR inhibition and triggers apoptosis, implying that the role of mTOR inhibitors may shift from cytostatic to cytotoxic [70]. Based on the finding that mTORC1-dependent lipid signaling drives YME1L-mediated proteolytic remodeling [71], rapamycin may indirectly modulate YME1L activity by inhibiting mTORC1, thereby affecting adaptive mitochondrial remodeling. For example, under metabolic stress such as hypoxia or amino acid starvation, the proteolytic activity of YME1L is enhanced, which broadly remodels the mitochondrial proteome to support cell survival [71]. Inhibition of mTORC1 by rapamycin may interfere with this process.

The pathogenesis of cardiovascular diseases (CVDs) is closely correlated with mitochondrial dysfunction, among which excessive production of mitochondrial ROS acts as a key driver of cellular injury, inflammatory responses, and cell death. Conventional antioxidants are difficult to target mitochondria specifically and exhibit limited clinical efficacy. Therefore, the development of mitochondria-targeted specific antioxidants has become an important strategy for CVD treatment. According to mechanisms of action and chemical structures, mitochondria-targeted antioxidants are mainly divided into two categories: triphenylphosphonium (TPP)-based derivatives and peptide compounds.

#### 4.1.1 TPP-Based Antioxidants

The targeting mechanism of these compounds relies on the negative inner mitochondrial membrane potential (Δψm) of the IMM. The lipophilic and cationic properties of TPP enable its massive accumulation across the concentration gradient on the inner mitochondrial membrane.

Mitoquinone (MitoQ): A conjugate of CoQ10 and the TPP carrier. After localizing to the inner mitochondrial membrane, it functions primarily as a lipophilic antioxidant, counteracting lipid peroxidation and scavenging harmful substances such as peroxynitrite.

10-(6′-plastoquinonyl) decyltriphenylphosphonium (SkQ1): Another lipophilic cation based on TPP, which carries plastoquinone analogs that specifically bind to cardiolipin, a signature phospholipid of the inner mitochondrial membrane. This binding is considered to stabilize cardiolipin and inhibit its detrimental interaction with cytochrome c, thereby preventing mitochondrial oxidative damage.

Mito-Tempo (MT): A TPP-conjugated piperidine nitroxide radical. As a mitochondrial superoxide dismutase mimetic, it mainly scavenges superoxide anion radicals and restricts hydroxyl radical generation.

#### 4.1.2 Peptide-Based Mitochondria-Targeted Antioxidants

SS-31 (Elamipretide): A highly permeable mitochondria-targeted antioxidant peptide whose targeting mechanism is independent of mitochondrial membrane potential, enabling it to exert effects even in functionally impaired mitochondria. Early studies attributed its protective effects to ROS scavenging capacity. Subsequent research revealed that SS-31 binds to cardiolipin and modulates the hydrophobic interaction between cardiolipin and cytochrome c, thereby inhibiting the peroxidase activity of cytochrome c and restoring its normal function in the electron transport chain.

YME1L is an essential protease located in the inner mitochondrial membrane. Loss of YME1L function directly induces dilated cardiomyopathy and heart failure in mice, which is associated with aberrant OPA1 processing and mitochondrial fragmentation [4]. Mitochondria-targeted antioxidants and regulatory strategies targeting the YME1L network theoretically possess potential synergistic therapeutic value based on shared pathophysiological foundations.

### 4.2 Biomarker-Guided Stratification for Targeting YME1L-Associated Pathways

Given that YME1L function exhibits context-dependent and tissue-specific characteristics, non-selective modulation of its activity lacks clinical feasibility, especially in aging-related cardiovascular diseases where mitochondrial reserve capacity is inherently compromised. Biomarker-guided patient stratification serves as an essential prerequisite for translating the YME1L pathway into clinical therapeutic applications.

YME1L expression level only provides preliminary reference value: high YME1L expression generally indicates poor prognosis in tumors, whereas low expression in aging cardiovascular diseases usually reflects the decline of mitochondrial resilience rather than pathological overactivation. A single expression level cannot reflect the functional status of the protease network.

Researchers established stable human embryonic kidney 293 (HEK293) cell lines with AFG3-like matrix AAA peptidase subunit 2 (AFG3L2) knockdown, YME1L knockdown, and combined AFG3L2/YME1L double knockdown, respectively. Cell counting, fluorescence imaging, and transmission electron microscopy were applied to analyze the effects of gene knockdown on cell proliferation, mitochondrial network morphology (degree of fragmentation), and cristae structure. Western blotting was used to detect the expression levels of key mitochondrial dynamic regulatory proteins, including long-form and short-form OPA1, metalloprotease OMA1, and the m-AAA complex subunit paraplegin (SPG7). The results demonstrated that the i-AAA protease YME1L constitutively cleaves OPA1, leading to the accumulation of both L-OPA1 and S-OPA1, which facilitates the maintenance of the mitochondrial fusion network [41]. In contrast, stress-induced cleavage of OPA1 by OMA1 causes the accumulation of soluble short-form OPA1, inhibits mitochondrial fusion, and induces mitochondrial fragmentation. AFG3L2-mediated initial cleavage of OMA1 plays a critical role in OMA1 maturation, and AFG3L2 deficiency results in a marked increase in the level of 60 kDa OMA1. These findings suggest that the OMA1/YME1L protein ratio can be used as a functional parameter to assess mitochondrial vulnerability in the future: an elevated ratio indicates excessive OPA1 cleavage, cristae destabilization, and mitochondrial fragmentation, serving as a characteristic marker of impaired stress adaptation in the aging myocardium. The OMA1/YME1L ratio has been well investigated in oncology studies. In osteosarcoma and non-small cell lung cancer, high YME1L mRNA expression is significantly correlated with adverse patient prognosis [72,73]. In a diethylnitrosamine-induced HCC model in male albino rats, hepatic OMA1 protein levels were reduced and YME1L levels were increased, with more pronounced changes at later experimental time points [74]. These preclinical observations require validation in human tissues before clinical biomarker applications can be considered. However, further studies are still needed to clarify the correlations between the OMA1/YME1L ratio and mitochondrial functional parameters (ROS production, ATP generation, redox homeostasis, and mitochondrial membrane potential) under conditions of myocardial stress and aging, so as to realize real-time evaluation of therapeutic intervention efficacy.

In clinical practice, constructing a YME1L-related biomarker stratification system by integrating protease network indicators, mitochondrial functional parameters, and clinical phenotypes (reduced exercise tolerance, diastolic dysfunction, and microvascular injury) enables precise screening of patient subpopulations that can benefit from mitochondria-targeted intervention strategies. This approach is of great significance for defining the therapeutic window, avoiding mitochondrial off-target toxicity, and promoting the clinical translation of basic research achievements.

### 4.3 Challenges and Future Directions of YME1L as a Therapeutic Target

Despite growing recognition of YME1L as a central regulator of mitochondrial quality control, several challenges must be addressed before its associated pathways can be translated into therapies for aging-related cardiovascular disease. Foremost among these is tissue specificity. YME1L exerts divergent effects across organs and cell types, reflecting differences in energetic demand, regenerative capacity, and stress exposure. Therapeutic strategies must therefore avoid systemic modulation that could compromise mitochondrial function in vulnerable tissues.

A second major challenge lies in distinguishing the effects of acute versus chronic modulation of YME1L activity. While short-term activation of mitochondrial stress responses may be adaptive, sustained perturbation of proteostasis could accelerate mitochondrial exhaustion in aging cardiomyocytes. Understanding the temporal dynamics of YME1L-mediated signaling will be essential for defining safe therapeutic windows.

Importantly, therapeutic enhancement of mitochondrial activity may not necessarily translate into improved cardiac resilience in aged myocardium. Excessive stimulation of oxidative metabolism could theoretically aggravate ROS accumulation and energetic instability under chronic stress conditions. Therefore, maintaining mitochondrial adaptability rather than simply augmenting bioenergetic output may represent a more appropriate therapeutic objective in cardiovascular aging.

In this context, YME1L should be viewed not as a conventional drug target but as a regulatory node within a broader mitochondrial resilience network. Future progress will depend on integrating mechanistic studies with precision medicine approaches to determine how, when, and in whom modulation of YME1L-associated pathways can meaningfully delay cardiovascular aging and disease progression.

## 5. Conclusion and Perspectives

Although substantial progress has been made in understanding the role of YME1L in mitochondrial quality control, several critical questions remain unresolved. It is still unclear whether YME1L decline represents a primary driver of cardiovascular aging or a secondary adaptive response to mitochondrial stress. Moreover, the tissue-specific consequences of YME1L modulation raise concerns regarding the safety of systemic therapeutic intervention. Future studies integrating spatial transcriptomics, mitochondrial proteomics, and longitudinal cardiovascular phenotyping will be essential to define when YME1L-associated pathways become maladaptive during aging progression. Clarifying these issues may facilitate the development of mitochondria-targeted therapeutic strategies for HFpEF and related age-associated cardiovascular disorders.
